# Effect of different palatal expanders with miniscrews in surgically assisted rapid palatal expansion: A non-linear finite element analysis

**DOI:** 10.1590/2177-6709.29.1.e2423195.oar

**Published:** 2024-03-04

**Authors:** Osman KOÇ, Nagihan KOÇ, Helder Baldi JACOB

**Affiliations:** 1 Yildiz Technical University, Department of Mechanical Engineering (Yildiz, Istanbul/Turkey).; 2 Independent researcher (Yildiz, Istanbul/Turkey).; 3 The University of Texas Health Science Center Houston School of Dentistry, Department of Orthodontics (Houston/TX, USA).

**Keywords:** Finite element analysis, Maxillary transverse deficiency, Palatal expansion, Surgically assisted rapid palatal expansion, Análise de elementos finitos, Deficiência transversal da maxila, Expansão palatina, Expansão rápida da maxila assistida cirurgicamente

## Abstract

**Introduction:**

Surgically assisted rapid palatal expansion (SARPE) has been the treatment of choice in subjects presenting skeletally mature sutures.

**Objective:**

The purpose of this study was to analyze stress distribution and displacement of the craniofacial and dentoalveolar structures resulting from three types of palatal expanders with surgical assistance using a non-linear finite element analysis.

**Material and Methods:**

Three different palatal expanders were designed: Model-I (tooth-bone-borne type containing four miniscrews), Model-II (tooth-bone-borne type containing two miniscrews), and Model-III (bone-borne type containing four miniscrews). A Le Fort I osteotomy was performed, and a total of 5.0 mm palatal expansion was simulated. Nonlinear analysis (three theory) method (geometric nonlinear theory, nonlinear contact theory, and nonlinear material methods) was used to evaluate stress and displacement of several craniofacial and dentoalveolar structures.

**Results:**

Regardless of the maxillary expander device type, surgically assisted rapid palatal expansion produces greater anterior maxillary expansion than posterior (ANS ranged from 2.675 mm to 3.444 mm, and PNS ranged from 0.522 mm to 1.721 mm); Model-I showed more parallel midpalatal suture opening pattern - PNS/ANS equal to 54%. In regards to ANS, Model-II (1.159 mm) and Model-III (1.000 mm) presented larger downward displacement than Model-I (0.343 mm). PNS displaced anteriorly more than ANS for all devices; Model-III presented the largest amount of forward displacement for PNS (1.147 mm) and ANS (1.064 mm). All three type of expanders showed similar dental displacement, and minimal craniofacial sutures separation. As expected, different maxillary expander designs produce different primary areas and levels of stresses (the bone-borne expander presented minimal stress at the teeth and the tooth-bone-borne expander with two miniscrews presented the highest).

**Conclusions:**

Based on this finite element method/finite element analysis, the results showed that different maxillary expander designs produce different primary areas and levels of stresses, minimal displacement of the craniofacial sutures, and different skeletal V-shape expansion.

## RESUMO

**Introdução:**

A expansão rápida da maxila assistida cirurgicamente (ERMAC) tem sido o tratamento de escolha em indivíduos que apresentam suturas esqueleticamente maduras.

**Objetivo:**

O objetivo deste estudo foi avaliar, utilizando uma análise não linear com elementos finitos, a distribuição de tensões e os deslocamentos das estruturas craniofaciais e dentoalveolares gerados por três tipos de expansores palatinos usados na ERMAC.

**Material e Métodos:**

Três tipos de expansores palatinos foram projetados: Modelo I (dento-osseossuportado com quatro mini-implantes), Modelo II (dento-osseossuportado com dois mini-implantes) e Modelo III (osseossuportado com quatro mini-implantes). Uma osteotomia Le Fort I foi realizada e foi simulada uma expansão palatina total de 5,0 mm. Um método de análise não linear (três teorias - teoria da não-linearidade geométrica, teoria do contato não linear e métodos para materiais não lineares) foi utilizado para avaliar a tensão e o deslocamento de diversas estruturas craniofaciais e dentoalveolares.

**Resultados:**

Independentemente do tipo de aparelho expansor palatino, a ERMAC produziu maior expansão anterior da maxila do que posterior (ENA variou de 2,675 mm a 3,444 mm e ENP variou de 0,522 mm a 1,721 mm); o Modelo I apresentou padrão de abertura mais paralela da sutura palatina mediana, com ENP/ENA igual a 54%. Com relação à ENA, o Modelo II (1,159 mm) e o Modelo III (1,000 mm) apresentaram maior deslocamento para baixo do que o Modelo I (0,343 mm). A ENP deslocou-se mais para anterior do que a ENA com todos os aparelhos; o Modelo III apresentou o maior deslocamento para anterior da ENP (1,147 mm) e da ENA (1,064 mm). Os três tipos de expansores apresentaram deslocamento dentário semelhante e separação mínima das suturas craniofaciais. Como esperado, diferentes *designs* de expansores palatinos produzem diferentes áreas primárias e níveis de tensões (o expansor osseossuportado apresentou tensão mínima nos dentes, e o expansor dento-osseossuportado com dois mini-implantes apresentou o maior).

**Conclusões:**

Com base nesse estudo de elementos finitos, os resultados mostraram que diferentes *designs* de expansores palatinos produzem diferentes áreas primárias e níveis de tensão, com deslocamento mínimo das suturas craniofaciais e diferentes expansões esqueléticas em forma de V.

## INTRODUCTION

Maxillary transverse deficiency is one of the most common dental problems, and it has been reported in 7% of the American children population increasing to 9.5% of the American adult population.^1^ To correct the transverse maxillary deficiency, maxillary expansion is a widely accepted procedure. Three treatment modalities are used today for maxillary expansion: rapid palatal expansion (RPE), slow palatal expansion (SPE), and surgically assisted rapid palatal expansion (SARPE). 

To effectively correct a skeletal maxillary transverse deficiency, the expansion must maximize the skeletal effects while minimize the dental ones. The maxillary expansion is the treatment of choice for growing subjects, but for skeletally mature subjects the RPE and SPE have limited orthopedic effects. The greatest skeletal response is observed before the calcification of the intermaxillary suture due to increased mechanical interlocking at maxillary articulations and the high cellular activity in the growing suture.^2^ ^-^ ^5^ Therefore, SARPE has been the treatment of choice in subjects presenting skeletally mature sutures.^6^ The surgical approach releases the palatal plates from the circumpalatal sutures and offers a true orthopedic maxillary expansion.^6^ ^,^ ^7^ Some surgical techniques have been proposed to mobilize the maxillary halves.^8^ ^-^ ^14^

Regardless of the technique, with or without surgery approach, various maxillary expander devices have been used to promote palatal expansion. These devices can be categorized as tooth-borne, bone-borne, tooth-tissue-borne, and hybrid (combination of two types). It is important for the clinician to choose the best design and position of the maxillary expander device for the most desirable outcome.^15^ ^,^ ^16^ Recently, the use of miniscrew assisted rapid palatal expansion (MARPE) has increased the maxillary expander design armamentarium, and successful maxillary expansion with these expanders has been reported with minimum side effects to teeth and periodontium.^17^ ^,^ ^18^ Although MARPE can promote midpalatal expansion in subjects presenting increased mechanical interlocking, SARPE is still preferred as the treatment in skeletally mature sutures due to predictability and stability.^19^ ^,^ ^20^

Various SARPE techniques were proposed, with the typical surgical procedure consisting of a Le Fort I osteotomy combined with corticotomy along the midpalatal suture. However, SARPE has shown inconsistencies in the midpalatal expansion pattern depending on the surgical technique and/or expander device design. The literature has reported more anterior dental expansion than posterior,^12^ ^,^ ^21^ approximately parallel,^12^ ^,^ ^22^ ^-^ ^24^ or more posterior than anterior dental expansion.^25^ Other complications such as asymmetric expansion between the right and left maxillary shelves have been reported.^20^ So, the dental and skeletal effects of different expanders and the stress distribution of the force used to promote maxillary expansion in the craniofacial structure still needs a better understanding.

It is known that the biomechanical response of bone under orthopedic forces, such as maxillary expansion, is complex. To study stresses, strains, and displacements, finite element method and finite element analysis (FEM/FEA) was introduced to medical field.^26^ This type of analysis had made it possible to accurately evaluate the biomechanical components of living structures in a non-invasive manner that can barely be measured *in vivo*.^27^ ^,^ ^28^ With the help of FEA, the amount of displacement occurring in the maxilla and the change in the stress occurring in the surrounding structures can be evaluated. In order to make FEM/FEA a clinically applicable tool, the image acquisition, the material properties, the model, and the analysis should be robust and accurate. In addition, a non-linear analysis is essential (non-linear FEM/FEA is more powerful algorithm and addresses problems that linear FEM/FEA does not).^28^ To closely mimic the *in-vivo* outcome, this study evaluates the stress, the strain, and the displacement of the craniofacial structures after a total of 5 mm activation under SARPE using non-linear solution.

The aim of this study was to compare the amount of displacement and stress distribution due to number of screws and different anchoring screw location of three different types of expansion appliance (bone-borne and hybrid) used in surgically assisted rapid palatal expansion on craniofacial and dentoalveolar structures using finite element analysis (FEA). The null hypothesis was that different expansion appliances do not change the pattern of maxillary expansion in a skeletally mature subject.

## MATERIAL AND METHODS

A finite element model was generated using an anatomical model of a 22-year-old adult male obtained from an anatomagraphic database developed from magnetic resonance imaging (MRI).^29^ Each image of the head featured a slice thickness of 2.00 mm (256 x 256 x 240 mm) and additional anatomical segmentations were introduced in the original data.^30^ ^,^ ^31^ The Committee for the Protection of Human Subjects (CPHS) of the University of Texas Health Science Center at Houston reviewed the submission and determined it does not meet the regulatory definition of human subjects research (HSC-DB-23-0712).

All head bones (cranial vault, cranial base, and facial bones) and teeth were in STL format downloaded and merged using ANSYS Workbench software (19.2, ANSYS Inc. Houston, PA, USA). For the finite element analysis (FEA), a symmetrical model of the craniofacial and maxillofacial structures with the maxilla, teeth, and periodontal ligaments was created independently and included in the overall model. The skull FEM model used in this study was constructed through the same processes as previous articles is similar.^32^ ^-^ ^34^ In the scenario of this study, the expansion device designs (bone-borne palatal expander and hybrid expander), the number of mini-screws, and the mini-screw placement site were different. 

Mimics software (version 15.0; Materialise, Leuven, Belgium) was used to edit and generate the final model. The offset command was used to model the periodontal ligament (PDL) with a thickness of 0.20 mm.^32^ Anisotropic materials were cortical and cancellous bone, periodontal ligament and homogeneous and isotropic materials were considered tooth and stainless steel. PDL was described by the hyperelastic material model - third order Ogden (Table 1).^32^ ^-^ ^34^

**Table 1:** Finite element model’s material properties.

Material properties	Elastic modulus (Mpa)	Poisson’s ratio	Shear modulus (Mpa)
Cortical Maxillary bone	Ex:12000	Vxy: 0.18	Gxy: 3600
	Ey: 11640	Vyz: 0.40	Gyz: 5400
	Ez: 15600	Vxz: 0.30	Gxz: 4100
Cortical Skull bone	Ex: 12580	Vxy: 0.48	Gxy: 4400
	Ey: 13600	Vyz: 0.24	Gyz: 6700
	Ez: 21200	Vxz: 0.32	Gxz: 4900
Cancellous bone	Ex:1148	Vxy: 0.32	Gxy: 434
	Ey: 1148	Vyz: 0.05	Gyz: 68
	Ez: 210	Vxz: 0.05	Gxz: 68
Periodontal Ligament	µ_1_: - 3420.83	µ_2_: 1434.35	µ_3_: - 5.56E-04
	α_1_: - 0.506	α_2_: - 0.134	α_3_: 13.708
	D_1_: 0 MPa^-1^	D_2_: 0 MPa^-1^	D_3_: 0 MPa^-1^
Dentin	19890	0.31	
Stainless steel	193000	0.35	

MPa: Megapascal. Ex, Ey, Ez: Elastic modulus in three directions. Vxz, Vyz, Vxz: Poisson’s ratio in three directions.

Gxy, Gyz, Gxz: Shear modulus in three directions. µ, α, D: Material parameters.

**Figure 1:**
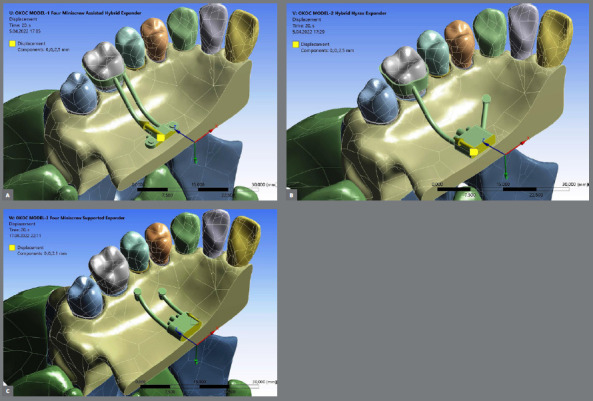
Three different palatal expander appliances design: Model-I is a tooth-bone-borne expander with four miniscrews placed 4 mm lateral to midpalatal suture (A), Model-II is a tooth-bone-borne with two miniscrews placed at the third rugae area (B), and Model-III is a bone-borne with four miniscrews placed 8 and 10 mm from the midpalatal suture (C). Screws were moved 2.5 mm in a transverse ( Z-axis ) direction ( 5 mm in total ).

Three different palatal expander appliances were designed (Fig 1). Model-I is a tooth-bone-borne palatal expander type containing four miniscrews placed at 4.00 mm lateral from the midpalatal suture and attached to maxillary first molars using double arm extension; the expander was located at the maxillary first molar level. Model-II is also a tooth-bone-borne palatal expander type but presenting only two miniscrews placed 6.00 mm from the midpalatal suture inserted at the third palatal rugae area, and it was attached to the first molar teeth by means of the appliance arms; this expander was also located at maxillary first molar level. Model-III is a bone-borne palatal expander type design presenting four miniscrew expander (two anterior miniscrews placed 6.00 mm and the two posterior miniscrews placed 8.00 mm from the midpalatal suture, respectively); the expander was located at maxillary second premolar level. For standardization, regardless of the palatal expander type, the miniscrews were modeled with ANSYS Workbench software presenting the same sizes (1.60 mm diameter and 10.00 mm length). After creating the structures and appliances, a LeFort I osteotomy without pterygomaxillary suture osteotomy was performed in all models. In the simulation of the palatal expansion, the expander was moved transversely 2.50 mm, implying a total of 5.00 mm. 

Several dental and skeletal landmarks were incorporated in this study. Dental landmarks were: central and lateral incisors, canine, first and second premolars, and first and second molars; skeletal landmarks were: anterior nasal spine (ANS), posterior nasal spine (PNS), frontomaxillary suture, zygomaticomaxillary suture, frontozygomatic suture, zygomatic arc, medial pterygoid plate, lateral pterygoid plate. The foramen magnum was accepted as the stable point as it was completely fixed and stable.^33^ Also, rotation and tipping of the maxilla were measured in all three planes.

### BOUNDARY CONDITIONS

The displacements of the aforementioned craniofacial structures were evaluated along X, Y, and Z coordinates against the transverse displacement of palatal shelves. The X-axis evaluated the changes in the anteroposterior plane, the Y-axis evaluated the changes in the vertical plane, and the Z-axis evaluated the changes in the transversal plane. Areas of stress were evaluated with the help of a different color scale band gap. Positive or negative values in the column of stress spectrum indicate tension or compression, respectively.

A time-dependent transient structural (dynamic) type of analysis was used to evaluate the Von Mises stress distribution and the amount of displacement. An analysis solution was performed using nonlinear geometric theory (large deformations), nonlinear contact theory (frictionless) and nonlinear material theory (anisotropic). By using the measurement probe, displacement and stress values were measured on the same element in all models with ANSYS Workbench software.

In all models, the displacement movement was transmitted to the maxilla with the help of miniscrews and the expansion screw was activated by 0.25 mm. In the symmetrical FEA model, transverse displacement of 2.50 mm was achieved in the Z-axis corresponding to 5.00 mm of movement. Results were obtained after a total activation of the expansion screw of 5 mm. Frictionless contact was defined between the maxillary surfaces separated by lateral osteotomy. Bonded contacts were defined between the teeth, the periodontal ligament and the screw of the expansion appliance and the maxilla. They move as a single unit, with no sliding or separation of faces and edges permitted. The amount of rotation of the maxilla was measured from angular changes made by the line passing through the nodes between anterior nasal spine (ANS) and posterior nasal spine (PNS) points with respect to the symmetrical plane of the skull.

### FINITE ELEMENT MODEL

Quadratic tetrahedral elements were used for volumetric mesh generation the symmetric model, only the expansion device and the anchor screw are divided into quadratic hexahedral elements mesh structure. The FEA model was composed of 2,186,123 nodes and 1,462,604 elements, and the maxilla contained more fine elements than elsewhere of the skull. The number of elements and nodes, and mesh size values were the same in all models, and the mean mesh skewness element quality convergence value was set to 0.16 (Table 2).^32^ ^-^ ^34^ The mesh structure is given in the figures where we share the displacement and stress results.

**Table 2:** Finite element model’s element, nodes, mesh size and average skewness element quality convergence values.

	Average skewness value	Nodes	Element	Mesh size (millimeters)	Mesh Element Type
Skull	0.19	1691902	1158657	2	Tetrahedral
Maxilla (inferior part)	0.17	105921	71404	1.3	Tetrahedral
Maxilla (superior part)	0.15	104642	71184	1.3	Tetrahedral
Periodontal ligament	0.40	95057	47053	0.6	Tetrahedral
Tooth	0.18	159921	106775	0.8	Tetrahedral
Palatal expansion appliance	0.46	28680	7531	0.4	Hexahedral

Mesh convergence was performed to validate the solution and to show that the measured values do not vary with element size. The best number of elements was established in the model for the mesh convergence. Mesh refinement was performed on the contact surface of the maxilla and sphenoid bone, in the area where the mini-screw was placed, and on the first molar tooth and its PDL.

## RESULTS

### AMOUNT OF DISPLACEMENT

**Figure 2:**
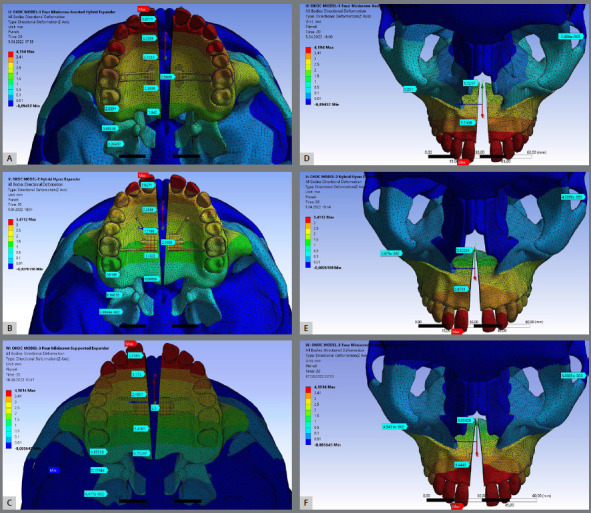
Displacement of the landmarks due to SARPE after 5 mm activation of the expander apparatus. Occlusal view simulation of the Model-I (A), Model-II (B), and Model -III (C), and frontal view simulation of the Model-I (D), Model-II (E), and Model -III (F).

With few exceptions, the anteroposterior movement direction of the landmarks was similar (Fig 2 and Table 3). Anterior teeth (central and lateral incisors) presented forward displacement while the posterior teeth showed a backward displacement. Central incisor of the Model-III presented the largest (0.520 mm) anteroposterior displacement followed by Model-II (0.121 mm) and Model-I (0.019 mm). Second molars moved distally more in Model-I (0.992 mm) with minimal posterior displacement for the Model-II and Model-III (0.852 mm and 0.802 mm, respectively). PNS moved anteriorly more than the ANS. Model-III presented highest amount of forward displacement for the PNS and ANS (1.147 mm and 1.064 mm, respectively), and the smallest anteroposterior movements were registered by the Model-I (0.153 mm and 0.175 mm for the PNS and ANS, respectively).

**Table 3:** Unilateral displacements (mm) of the evaluated landmarks after 5 mm of the maxillary expander activation.

	Model-I			Model-II			Model-III		
Anatomical structure	X-Axis	Y-Axis	Z-Axis	X-Axis	Y-Axis	Z-Axis	X-Axis	Y-Axis	Z-Axis
Central incisor	0.019	-0.269	4.194	0.121	-1.147	3.411	0.520	-0.983	4.361
Lateral incisor	-0.478	0.026	4.087	-0.252	-0.933	3.230	-0.010	-0.731	4.173
Canine	-0.692	0.273	3.843	-0.520	-0.716	2.923	-0.379	-0.535	3.809
First premolar	-0.809	0.488	3.570	-0.711	-0.505	2.563	-0.581	-0.370	3.285
Second premolar	-0.908	0.663	3.344	-0.857	-0.335	2.236	-0.765	-0.206	2.860
First molar	-0.958	0.820	3.137	-0.853	-0.174	2.048	-0.849	-0.114	2.400
Second molar	-0.992	0.904	2.668	-0.852	-0.037	1.315	-0.802	-0.008	1.681
Anterior nasal spine	0.153	-0.343	3.210	0.669	-1.159	2.675	1.064	-1.000	3.444
Posterior nasal spine	0.175	-0.080	1.721	0.730	-0.498	0.522	1.147	-0.544	0.684
Frontomaxillary suture	-0.481	1.025	0.001	-0.018	0.014	0.000	-0.217	0.046	0.000
Zygomaticomaxillary suture	0.067	1.173	0.201	0.011	0.044	0.036	0.027	0.085	0.045
Frontozygomatic suture	-0.592	1.005	0.031	-0.026	0.026	0.005	-0.035	0.046	0.004
Zygomatic arch	-0.187	0.644	-0.007	-0.017	0.014	0.004	-0.015	0.023	0.005
Medial pterygoid plate	-0.385	0.847	0.896	-0.153	0.025	0.140	-0.101	0.040	0.153
Lateral pterygoid plate	-0.297	0.618	0.216	-0.075	0.040	0.038	-0.049	0.037	0.044
Foramen magnum	0.000	0.000	0.000	0.000	0.000	0.000	0.000	0.000	0.000
Angle of rotation and tipping	0.37°	1.83°	-1.93°	0.84°	1.19°	-2.78°	0.65°	1.27°	-3.70°

X-axis, anteroposterior plane; Y-axis, vertical plane; Z-axis, transverse plane. Positive (+) values indicate forward, outward or upward displacement, and negative (-) values indicate backward, inward or downward displacement. Median and lateral osteotomies were made in all models without pterygomaxillary suture separation.

In regards to facial sutures displacements, the largest anteroposterior displacements were registered at the frontozygomatic and frontomaxillary sutures of the Model-I (posterior displacement of 0.592 mm and 0.481 mm, respectively). Although zygomaticomaxillary suture showed some anteroposterior changes due to SARPE, the amount of forward movement was minimal (ranging from 0.011 mm to 0.067 mm).

Vertical displacement of the landmarks was seen due to SARPE (Fig 2 and Table 3). Model-I presented downward movement of the central incisor (0.269 mm) and progressively upward movement from lateral incisor (0.026 mm) to second molars (0.904 mm), while Model-II and Model-III presented downward displacement for all evaluated teeth. PNS showed similar amount of displacement for Model-II and Model-III (0.498 mm and 0.544 mm, respectively) while Model-I presented the smallest (0.080 mm). In regards to ANS, Model-II (1.159 mm) and Model-III (1.000 mm) presented larger downward displacement than Model-I (0.343 mm). Facial sutures and bones moved slightly upwards. Model-I showed the largest movements (ranging from 1.005 mm to 1.173 mm, respectively for frontozygomatic and zygomaticomaxillary sutures). Facial sutures of Model-II and Model-III presented less than 10% of the amount of the Model-I.

Transversely, all models present lateral movements of the landmarks (Fig 2 and Table 3). As expected, anterior dental areas showed larger amounts of separation than posterior dental areas. Model-I and Model-III experienced over four mm (4.194 mm and 4.361 mm, respectively) of transverse movement for the central incisors whereas Model-II experienced 3.411 mm. Second molars presented more transverse movements on Model-I (2.668 mm) followed by Model-III (1.681 mm), and Model-II (1.315 mm). ANS also separated more than PNS, with Model-I presenting more parallel separation. Craniofacial sutures showed minimal transverse changes; zygomaticomaxillary suture showed the highest values ranging from 0.036 mm (Model-II) to 0.201 mm (Model-I). The medial pterygoid plate showed the highest amount of transverse displacement (0.896 mm for Model-I, 0.153 mm for Model-III, and 0.140 mm for Model-II) than the lateral pterygoid plate (0.216 mm for Model-I, 0.044 mm for Model-III, and 0.038 mm for Model-II). 

In all models, rotation and tipping of the maxilla were observed during expansion (Table 3). The wedge-shaped expansion pattern was observed in all models, but it was more prominent in Model-II and Model-III (Fig 2). Sagittally, the highest values were observed in the Model-II (0.84°) followed by Model-III (0.65°) and Model-I (0.37°). Vertically, Model-I presented the highest (1.83°) amount of rotation while Models I and II presented less vertical rotation (1.19° and 1.27°, respectively). Transversally, Model-III showed the largest amount of rotation (3.70°) followed by Model-II (2.78°) and Model-I (1.93°). 

### STRESS DISTRIBUTION

**Figure 3:**
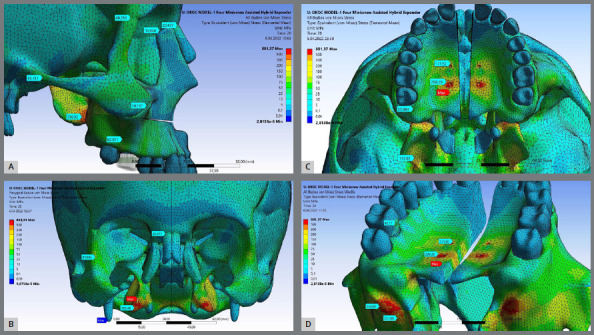
Stress distribution values for craniofacial structures including the miniscrew sites for Model-I expander type after 5 mm activation of the expander apparatus. (A) Sagittal view, (B) Coronal slice view showing the pterygomaxillary suture, (C) Axial view, and (D) Axo-posterior view. Highest stress area is presented in red.

**Figure 4:**
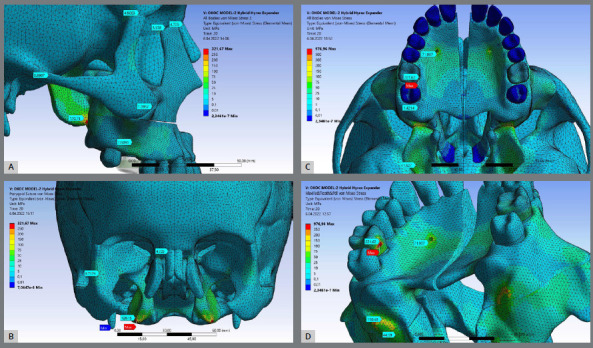
Stress distribution values for craniofacial structures including the miniscrew sites for Model-II expander type after 5 mm activation of the expander apparatus. (A) Sagittal view, (B) Coronal slice view showing the pterygomaxillary suture, (C) Axial view, and (D) Axo-posterior view. Highest stress area is presented in red.

**Figure 5:**
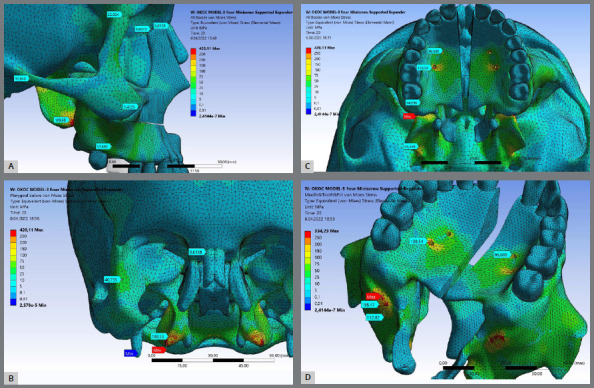
Stress distribution values for craniofacial structures including the miniscrew sites for Model-III expander type after 5 mm activation of the expander apparatus. (A) Sagittal view, (B) Coronal slice view showing the pterygomaxillary suture, (C) Axial view, and (D) Axo-posterior view. Highest stress area is presented in red.

Model-I (Fig 3) showed the highest stress distribution value for all craniofacial structures followed by Model-II (Fig 4) while the Model-III (Fig 5) generally showed the lowest stress distribution value (Table 4). Model I (Fig 3) and Model III (Fig 5) showed the highest stress patterns at the medial pterygoid plate (350.3 MPa and 189.60 MPa, respectively) followed by the miniscrews area (299.3 MPa and 138.5 MPa, respectively). Interesting, Model-II showed that first molars received the highest stress pattern (221.6 MPa) followed by the medial pterygoid plate (120.10 MPa). As expected, Model-III showed approximately zero stress at first molar.

**Table 4:** The mean elemental stress (von Mises stress and first principal stress) distribution of the evaluated structures after 5 mm of the maxillary expander activation.

	Model-I		Model-II		Model-III	
Structure	Von Mises Stress (MPa)	First Principal Stress (MPa)	Von Mises Stress (MPa)	First Principal Stress (MPa)	Von Mises Stress (MPa)	First Principal Stress (MPa)
Frontomaxillary suture	10.93	12.90	3.38	3.09	5.69	3.57
Zygomaticomaxillary suture	14.19	4.37	1.40	0.06	5.42	1.61
Frontozygomatic suture	49.25	-2.31	4.81	-0.18	22.00	-1.09
Zygomatic arch	35.13	28.35	0.89	0.14	15.94	9.47
Nasal frontal suture	20.47	18.70	4.73	5.47	8.61	9.78
Medial pterygoid plate	350.30	79.66	120.10	20.18	189.60	42.67
Lateral pterygoid plate	153.60	35.37	41.82	6.43	84.44	19.42
Maxillary tuberosity	35.98	21.91	1.42	1.29	14.69	3.42
Posterior nasal spine	2.28	0.23	0.02	0.05	0.33	0.01
Anterior nasal spine	0.04	0.22	0.02	0.02	0.01	0.25
Miniscrew region	299.30	172.10	71.90	55.97	138.50	97.90
First molar	40.77	11.86	221.60	28.93	0.02	0.02

Positive (+) values indicate compression stress and negative (-) values indicate tensile stress; MPa=megapascal.

It is important to notice that PNS and ANS presented the lowest von Mises stress values of the evaluated skeletal landmarks (Table 4). PNS stress distribution ranged from 0.015 MPa (Model-II) to 2.280 MPa (Model-I). ANS showed stress distribution values below 0.050 MPa for all the models, ranging from 0.002 MPa (Model-III to 0.043 MPa (Model-I). 

### STRUCTURAL ERROR AND SINGULARITY

Maximum stress element (MSE) mean values were high: Model-I presented the highest MSE value (881 MPa) at the area of the most distal miniscrew (Fig 3C), Model-II showed the highest MSE value (976MPa) at lingual surface of the first molar (Fig 4C), and the Model-III showed the highest MSE values (282MPa) at the pterygomaxillary junction (Fig 5C). The red colored regions in the figures show the elements with the highest stress values and structural errors. When these areas presenting the highest stress are examined in the models, it means the stress accumulates at the nodal points and in several elements of that volume. To optimize the accuracy of the solution and the analysis time, mesh convergence was performed in the maximum stress regions. According to the mesh convergence results, it was observed that the elemental average stress value measured in the maximum stress region did not change more than 6%, indicating that an optimum number of elements has been determined for the model.

## DISCUSSION

Regardless of the maxillary expander device type, SARPE produces greater anterior maxillary expansion than posterior. Although the V-shaped opening shape, the appliance design influences the skeletal and dental maxillary expansion pattern. Model-I showed less accentuate and the Model-II more accentuate V-shaped (PNS/ANS proportion of 54% and 20%, respectively). The skeletal V-shaped expansion pattern has been reported in other FEM/FEA studies regardless of the osteotomy technique.^12^ ^,^ ^21^ Clinical studies have also reported a V-shaped opening of the midpalatal suture due to SARPE.^13^ ^,^ ^24^ ^,^ ^36^ ^,^ ^37^ Comparisons are problematic due to study design, surgical technique, and appliance design and position. In addition, it has been shown that age is also directly related to the opening pattern of the midpalatal suture, with older subjects presenting smaller and lesser parallel maxillary expansion.^38^ The anteroposterior position of the expander device can also influence the maxillary V-shape expansion.^15^ ^,^ ^21^ It is important for the clinician to choose the best design and position of the maxillary expander device according to the desirable outcome.^15^

SARPE produces vertical skeletal displacement of the maxilla. Based on ANS and PNS, SARPE displaced the maxilla downward more in the anterior than in the posterior. The more pronounced inferior than superior displacement of the maxillary complex was previously reported before in FEM/FEA and clinical studies, regardless of the surgery technique.^12^ ^,^ ^13^ ^,^ ^36^ The pattern of maxillary expansion and displacement after SARPE is probably due to the remaining attachments of the maxilla superiorly to the craniofacial bones, which produce a greater amount of resistance against expansion at the upper segments of the maxilla.^39^

In general, SARPE provides a similar pattern of dental displacement regardless of the expander device design. Dental structures such as central incisors presented downward and more lateral displacements, whereas the structures such as molars presented upward and less lateral displacement. Dental displacement pattern due to SARPE has shown inconsistencies according to surgical technique and/or expander device design, and more anterior dental expansion than posterior,^12^ ^,^ ^21^ approximately parallel,^12^ ^,^ ^22^ ^-^ ^24^ and more posterior than anterior dental expansion,^25^ have been reported by the literature. In addition, the V-shaped separation of the maxillary halves provides teeth displacement to not follow the same amount or direction of the skeletal base. The literature has demonstrated rotation of the maxillary segments after SARPE with or without pterygomaxillary distraction.^19^ ^,^ ^40^ The area closer to the fulcrum experiences less expansion, and the hemimaxillae are displaced transversely, with more expansion of the inferior part.^40^

Maxillary expansion due to SARPE produces minimal displacement of the craniofacial sutures. Most (81%) of the craniofacial suture measurements presented sutural displacement smaller than 0.5 mm. The largest craniofacial sutural displacement was promoted by the Model-I type device (slightly over 1.0 mm of vertical displacement of the frontomaxillary, zygomaticomaxillary, and frontozygomatic sutures). Rapid palatal expansion with and without surgical assistance have also shown minimal displacement of craniofacial sutures.^41^ It has been suggested that the resistance of transverse maxillary expansion is the zygomaticomaxillary buttress and the pterygomaxillary junction,^19^ and eliminating the resistance to lateral movement by osteotomy should allow for larger maxillary basal bone movement.^43^

Maxillary expander design influences the transversal displacement of medial and lateral pterygoid plates. Maxillary expanders more posteriorly inserted, such as Model-I, showed considerably more vertical and lateral displacement of the medial and lateral pterygoid plates. Literature has shown maxillary expansion resistance by the pterygoid plate,^44^ but the displacement of the pterygoid plates or zygomatic buttress has been controversial. Again, comparisons are problematic because the studies design, but the anteroposterior position of the dental and/or skeletal anchorage has influence on pterygoid plates.^15^ ^,^ ^41^ ^,^ ^45^ ^,^ ^46^ In growing subjects, minimal displacement of the pterygoid plates has been reported using FEM/FEA.^47^ It seems that the surgical technique could play important role on pterygoid plates displacement,^39^ ^,^ ^48^ ^,^ ^49^ but surgeons are concerned about of the advantages of the pterygomaxillary disjunction due to the increasing of fractures in adjacent bones or injuring the vasculature in the posterior of the maxilla.^22^

Different maxillary expander designs produce different primary areas and levels of stresses. As expected, the bone-borne device presented minimal stress at the teeth (maxillary first molars) and the tooth-bone-borne two miniscrew expander device presented the highest stress level on the anchor teeth. Medial and lateral pterygoid plates are the skeletal area presenting the highest level of stress, with the Model-I presented approximately twice and thrice the stress of the Model-III and Model-II, respectively. Previous studies reported high level of stress at midpalatal and pterygomaxillary sutures.^12^ ^,^ ^32^ ^-^ ^34^ ^,^ ^41^ ^,^ ^50^ In general, craniofacial sutures stress dissipation follows from outside to inside and from superior to inferior with the structures connected to the cranium base exhibiting more stress level,^45^ and our study corroborates in showing similar trend with very few exceptions. Some authors mentioned that the osteotomies are more important in reducing stress in certain areas than the type of the device.^48^ ^,^ ^49^ In this study we did not compare osteotomy techniques, but the literature has shown that additional pterygomaxillary junction release procedure reduces stress near cranial base and/or anchor teeth.^33^ ^,^ ^41^ ^,^ ^48^ ^,^ ^51^

Finite element model and analysis is a standard engineering tool used to precisely assess local stress/strain/displacement in geometrically intricate structures such as craniofacial complex. It is also an ideal simulation method for evaluating clinical problems. FEM/FEA includes numerous simplifications and assumptions, which can decrease the accuracy of the analysis and geometric model - boundary conditions and mesh structure are the factors affecting the solution results. Conversely to our study, some studies did not fully model the craniofacial structure nor the anchor miniscrew of the maxillary expander device.^21^ ^,^ ^37^ ^,^ ^40^ ^,^ ^48^ ^-^ ^51^ Also, it is important to mention the boundary condition between bone, PDL, tooth, and expansion appliance, which some studies did not mentioned. ^21^ ^,^ ^37^ ^,^ ^40^ ^,^ ^48^ ^-^ ^51^

In this study, the miniscrews were placed in safe and stable areas of the palate determined by clinical studies. As aforementioned, several factors such as patient’s age, gender, type of expansion appliance design, miniscrew placement site, mid palatal suture maturity, bone density, and the response of muscles and soft tissues may affect the amount of expansion and success rate. Some of these factors (boundary conditions) could not be included in the analysis process in FEA studies.^32^ ^-^ ^34^

An important consideration in FEA studies is the reliability of the results and the repeatability of the analysis. Among other factors such as boundary conditions (amount of displacement, type of contact, and applied forces) and material properties, the determination of mesh structure (number of elements and nodes) and mesh convergence (which refers to the sufficient number of elements that must be present in the model) are very important for the repeatability of the results in the analysis.^32^ ^-^ ^34^ ^,^ ^52^ ^,^ ^54^ The mesh structure has a direct impact on the accuracy of the solution and the results. The number of elements required in the model is found by mesh convergence. According to the spectrum of mesh metrics generated by ANSYS Software, mesh convergence is necessary to produce reliable results. ^32^ ^-^ ^34^ ^,^ ^52^ ^,^ ^56^ In the analysis, it is not desired that the results change with changes in the mesh size.

**Figure 6:**
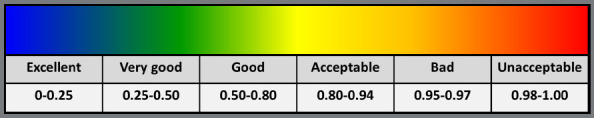
Skewness mesh metrics spectrum.

The skewness mesh metrics spectrum (Fig 6) is important for a better understanding of the study’s mesh quality. Low orthogonal quality and high skewness values are not recommended;^55^ these values influence the accuracy of the analysis results, and the accuracy decreases as the average skewness value progresses from excellent to good. The mean mesh convergence value and skewness element quality of our model were excellent for all parts that make up the geometry. Previous FEM/FEA studies used incomplete and no-smooth geometric models, and did not specify mesh, mesh convergence, element size, number of nodes, boundary conditions, and expansion appliance type.^35^ ^,^ ^49^ ^-^ ^51^ ^,^ ^53^ So, comparison is problematic and carefully attention to the previous literature results should be a must.

A protocol and nonlinear analysis method similar to the clinical activation of the screw is recommended to obtain more reliable and accurate results in FEM/FEA studies.^41^ ^,^ ^48^ Some previous FEM/FEA studies simulating SARPE only evaluated the initial activation of the expansion screw (0.5-1.00 mm) unlike clinical application.^41^ ^,^ ^56^ To better reflect the clinical situation, a total of 5.00 mm (20 activation of the expander screw) widening was performed in a symmetrical model in our study. There are few FEM/FEA studies about SARPE that moved the expansion appliance screw directly, totaling 5 mm, in a simulation of bone-born expansion without pterygomaxillary sutures (PTMS).

Maxillary expander device design can alter the highest stress site under surgically assisted palatal expansion. Four-miniscrew expander devices (Model-I and Model-III) generated highest stress at the medial pterygoid plate region, while the two-miniscrew expander device (Model-II) produced the highest stress at the first molar level. Using similar skull model and FEA/FEM, but using only bone-borne expanders with a slightly different miniscrew position, Koç et al.^32^ showed the stress value in the screw placement region, medial and lateral pterygoid plate was low in the model in which the expansion appliance anchor miniscrew was placed closer to the alveolar bone. As expected our Model-III presented similar results to aforementioned study with the lowest stress value measured for the same regions due to similarity of the skeletal anchorage and expander designs. In addition, the displacement of the central incisor, ANS, and PNS were very similar to our study. Comparing SARPE with and without pterygomaxillary sutures (PMS) osteotomy, Koç and Jacob^33^ showed that in the palatal expander presenting miniscrew closer to the midpalatal suture, the highest stress values were measured in the medial pterygoid plate, screw placement region and lateral pterygoid plate, respectively. The highest stress value for the same regions in our scenario was measured in Model-I and showed approximately the same stress value. Unlike previous studies, we examined the clinical results of different types of (tooth-born, bone-born and hybrid) expansion appliance and anchor screw positions.

With the exception of the miniscrew area, the highest stress levels were measured in the medial and lateral pterygoid plates. Stress and displacement values measured from the craniofacial and maxillofacial sutures were found to be relatively similar in studies employing FEA to simulate bone-supported expansion in SARPE without PTMS.^32^ ^-^ ^34^ ^,^ ^56^ The reason why the results differ depends on the type of expansion appliance, the miniscrew anchorage position, the accuracy of the model used and the mesh structure (quality).^32^ ^-^ ^34^ ^,^ ^57^

The maxillary palatal expander appliance Model-I moved the anterior region of the maxilla and the teeth very slightly anteriorly and downward, and the teeth in a more horizontally axis (in the transversal direction). The posterior region of the maxilla moves less horizontally (transversely) and more upwards. It also forces rotation and tipping over (counterclockwise) in the posterior (zygomatic bone) region. Therefore, higher stress values occur in the screw, medial and lateral pterygoid plate area due to resistance as a result of movement in the maxilla and pterygomaxillary suture contact area. The expansion appliance used in Model-II and Model-III moves the anterior and posterior region of the maxilla and the teeth horizontally direction (transversely) and more forward and downward than in the Model-I. A very small amount of rotation and tipping over movement occurs in the maxilla and teeth, while makes more translational movement in the lateral (transversally) direction. Due to less contact and resistance in the pterygomaxillary suture area, lower stress values occur in the screw, medial and lateral pterygoid plate area.

### CLINICAL IMPLICATIONS

SARPE produces greater anterior maxillary expansion than posterior expansion, regardless of the maxillary expander design, but the V-shaped expansion is less noticeable for the four mini-screw tooth-borne expander design (Model-I). In addition, the four mini-screw expander design presents more rotation/tilting (in the posterior, zygomatic bone region) and more transverse movement of the maxilla. The expander type can be recommended according to the required maxillary expansion pattern and displacement.

The impact of SARPE on craniofacial sutures is minimal. Although the amount and pattern of maxillary expansion are comparable, Model-I creates the maximum stress values than Model-III in all the evaluated sutures, which is important in selecting this type of maxillary expansion appliance. Clinically, maxillary expansion has been reported to be more predictable under bone-borne maxillary expanders than tooth-borne expanders in growing patients.^58^ In addition, when compared to multipiece Le Fort, SARPE produce less skeletal changes.^59^

Stress on teeth can be associated to tipping, which can lead to potential tooth and surrounding tissue damage, and pain response. Ideally, maxillary expansion should maximize dentofacial orthopedics with minimal orthodontic tooth movement. To avoid high stress on teeth, orthodontists should make use of bone-borne (Model-III) or tooth-bone-borne with four miniscrews (Model-I), which present not only more skeletal anchorage but also more posterior miniscrews (closer to the maxillary center of resistance). When periodontal health is doubtful, the bone-borne maxillary expander (Model-III) should be elected.

These SARPE-FEM/FEA simulation results may be helpful and instructive for the clinical application, but in-vivo studies are required to confirm these results.

### LIMITATIONS

Clinical conditions are difficult to apply and standardize on a single patient with the same clinical characteristics as the patient who was used to generated the FEM/FEA for this study. Additionally, the resistance of tooth movement may vary as the root approaches the cortical bone, and some areas of stress could be different. In this FEA simulation, relapse after expansion, bone remodeling, effects of soft tissues, muscles and chewing forces were not included in the analysis. The accuracy of the simulation should increase if these factors could be added to the boundary conditions. Therefore, the displacement and stress values may be higher than the clinical values and our findings could differ from clinical results. The highest stress value observed were in the pterygomaxillary suture and mini-screw insertion areas, and the maximum stress values occurring were extremely high. These values, which were a function of the FEM/FEA process (in a few elements of the volume and nodes components of the pterygomaxillary suture and miniscrew region) did not affect the results. In areas with sharp corners high stress values occur due to the mathematical calculation errors in FEA.^32^ ^-^ ^34^ ^,^ ^55^ ^,^ ^57^ In addition, the maximum stress value does not actually accumulate at a single point as in the analysis result, due to the flexibility of the bone against loads and its self-healing feature.^32^ ^-^ ^34^ ^,^ ^55^ ^,^ ^57^

## CONCLUSIONS

Based on finite element method/finite element analysis, the results of this surgical assisted rapid palatal expansion study reject the null hypothesis because there is a slightly different maxillary expansion pattern in a skeletally mature subject due to expander device design. In addition, the following conclusions can be drawn:

» SARPE produces greater anterior than posterior maxillary expansion pattern regardless of the maxillary expander design, but the V-shaped expansion is less noticeable with the four mini-screw tooth-borne expander design.
» There is a vertical skeletal displacement of the maxilla due to SARPE.
» Maxillary expansion with to SARPE produces minimal displacement of the craniofacial sutures.
» Different maxillary expander designs produce different primary area and level of stresses.
» With the exception of the miniscrew area, the highest stress levels were measured in the medial and lateral pterygoid plates.
